# Novel insights into surfactant protein C trafficking revealed through the study of a pathogenic mutant

**DOI:** 10.1183/13993003.00267-2021

**Published:** 2022-01-27

**Authors:** Jennifer A. Dickens, Eimear N. Rutherford, Susana Abreu, Joseph E. Chambers, Matthew O. Ellis, Annemarie van Schadewijk, Pieter S. Hiemstra, Stefan J. Marciniak

**Affiliations:** 1 Cambridge Institute for Medical Research, Cambridge, UK; 2 Dept of Pulmonology, Leiden University Medical Center, Leiden, The Netherlands

## Abstract

**Background:**

Alveolar epithelial cell dysfunction plays an important role in the pathogenesis of idiopathic pulmonary fibrosis (IPF), but remains incompletely understood. Some monogenic forms of pulmonary fibrosis are associated with expression of mutant surfactant protein C (SFTPC). The commonest pathogenic mutant, I73T, mislocalises to the alveolar epithelial cell plasma membrane and displays a toxic gain of function. Because the mechanisms explaining the link between this mutant and IPF are incompletely understood, we sought to interrogate SFTPC trafficking in health and disease to understand the functional significance of SFTPC-I73T relocalisation.

**Methods:**

We performed mechanistic analysis of SFTPC trafficking in a cell model that reproduces the *in vivo* phenotype and validated findings in human primary alveolar organoids.

**Results:**

We show that wild-type SFTPC takes an unexpected indirect trafficking route *via* the plasma membrane and undergoes the first of multiple cleavage events before reaching the multivesicular body (MVB) for further processing. SFTPC-I73T takes this same route, but its progress is retarded both at the cell surface and due to failure of trafficking into the MVB. Unable to undergo onward trafficking, it is recycled to the plasma membrane as a partially cleaved intermediate.

**Conclusion:**

These data show for the first time that all SFTPC transits the cell surface during normal trafficking, and the I73T mutation accumulates at the cell surface through both retarded trafficking and active recycling. This understanding of normal SFTPC trafficking and how the I73T mutant disturbs it provides novel insight into SFTPC biology in health and disease, and in the contribution of the SFTPC mutant to IPF development.

## Abstract 2

**Studying how SFTPC mutations cause familial pulmonary fibrosis may help us develop new therapies for IPF. By studying a pathogenic mutation, this study reveals new insights into SFTPC handling in alveolar cells and how this is perturbed in disease.** https://bit.ly/344VUhm

## Introduction

In idiopathic pulmonary fibrosis (IPF), pathological scarring of the lung impairs gas exchange to cause premature death [1]. Alveolar type 2 (AT2) epithelial cells, the cells that synthesise surfactant and are the progenitors of AT1 cells [2], are crucial in IPF development. It is increasingly recognised that AT2 injury and dysfunction in IPF trigger pathological lung remodelling [3], but limited understanding of the initiating events in IPF has hampered development of therapies that directly modify early pathogenic mechanisms. Familial pulmonary fibrosis (FPF), caused by mutations of individual genes, offers a unique opportunity to examine how specific AT2 defects cause pulmonary fibrosis [4].

Autosomal dominant mutations in surfactant protein C (SFTPC), which is expressed exclusively in AT2 cells, cause FPF [5]. Expression of the commonest pathogenic variant, SFTPC-I73T [6], can manifest as FPF in childhood or in adults [6, 7]. Some of these variants of SFTPC do not traffic beyond the endoplasmic reticulum (ER) because they are unable to fold correctly. This results in “ER stress” [8], which is seen in the lungs in FPF and sporadic IPF [9]. However, SFTPC-I73T does not cause ER stress, but instead has been reported to mislocalise to the cell surface by unknown mechanisms [10].

**FIGURE 1:**
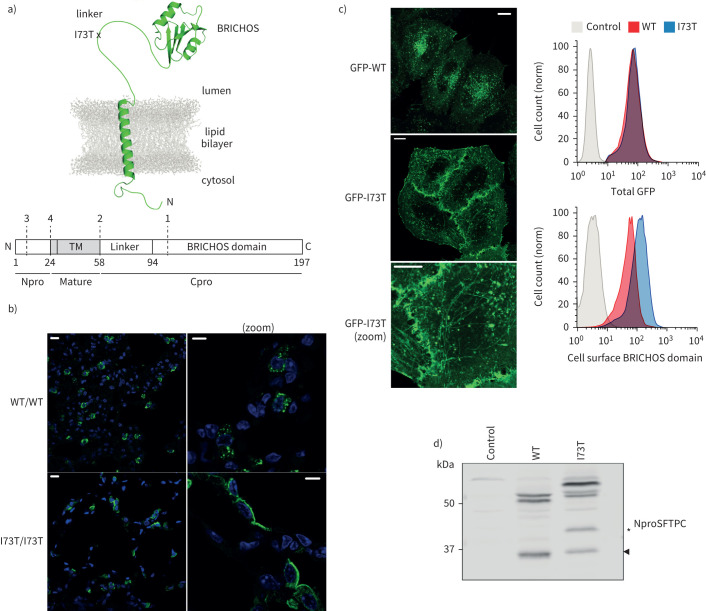
Surfactant protein C (SFTPC)-I73T is aberrantly located and processed *in vivo* and *in vitro*. a) Schematics of SFTPC delineating structure and location of the pathogenic I73T mutation along with domains and approximate reported cleavage sites (dashed lines, numbered 1–4). b) ProSFTPC immunostaining of alveolar tissue from transgenic mice conditionally expressing SFTPC-WT (wild type) or SFTPC-I73T reveal punctate intracellular staining of SFTPC-WT and redistribution of SFTPC-I73T to the apical plasma membrane. c) When green fluorescent protein (GFP)-SFTPC-WT is expressed in HeLa cells it localises in intracellular punctate structures, whereas GFP-SFTPC-I73T relocalises to the plasma membrane and intracellular tubular structures. In cells expressing equal amounts of GFP (upper right panel), there is an excess of cell surface SFTPC-I73T as measured by a BRICHOS domain antibody (lower right panel). d) Immunoblotting of lysates from vector control or GFP-SFTPC expressing cells using an NproSFTPC antibody reveal altered processing of SFTPC-I73T. TM: transmembrane. Scale bars=10 μm or 5 μm (zoomed images).

Understanding SFTPC trafficking is necessary if we are to explain how the FPF-causing SFTPC-I73T variant causes protein mislocalisation and AT2 dysfunction. SFTPC is produced as a proprotein (proSFTPC) which undergoes several proteolytic steps before being secreted, although surprisingly the location and precise nature of the maturation steps remain unclear. ProSFTPC is a type 2 transmembrane protein that comprises four domains (figure 1a): 1) an N-terminal cytosolic domain required for post-Golgi targeting [11–14]; 2) a transmembrane helix that forms the mature SFTPC protein [15]; 3) an unstructured linker domain; and 4) a C-terminal BRICHOS domain that ensures correct protein folding. Current models suggest that proSFTPC traffics directly from early cellular compartments of the secretory pathway to specialised late endosomes called multivesicular bodies (MVBs), and then to AT2-specific lamellar bodies prior to secretion [16]. *En route*, sequential C- then N-terminal cleavages yield mature SFTPC (figure 1a) [17]. However, the I73T mutation disrupts this process resulting in abnormal accumulation of immature SFTPC at the plasma membrane and in early endosomes [10, 18]. This is reported to have toxic effects on AT2 function, perturbing phospholipid uptake and protein degradation *via* autophagy and mitophagy [18, 19]. Understanding how the I73T mutation prevents normal trafficking of SFTPC may ultimately enable the development of therapeutics that may prevent AT2 injury and dysfunction, and prevent the onset of fibrosis.

Here, we present a detailed analysis of SFTPC trafficking that shows for the first time that both wild-type and I73T-mutant SFTPC unexpectedly traffic *via* the plasma membrane. Mislocalisation of SFTPC-I73T results from aberrant recycling of this protein to the cell surface owing to its failure to targeted to the MVB, resulting in its accumulation at the cell surface and recycling endosomes. Our investigation of a rare pathogenic variant thus provides fundamental insights into the biology of SFTPC and the mechanism of disease.

## Materials and methods

Detailed methods including details of chemicals, antibodies, cell lines, small interfering (si)RNAs, CRISPR guides, creation of expression vectors and cell-based assays are available in the supplementary material.

### Cell culture

HeLa cells were maintained in DMEM+10% fetal bovine serum (+400 µg·mL^−1^ geneticin for stable cell lines). Transfection of DNA was achieved using FuGene 6 (Promega) and clonal transgenic cell lines generated by selection and single-cell sorting. Knockout cell pools were generated by liposomal transfection with vectors expressing Cas9, guide RNA, and a fluorescent marker used for fluorescence-activated cell sorting.

### Organoid culture

Alveolar organoids were obtained using a feeder-free organoid-based expansion method for tissue-isolated primary alveolar cells. A manuscript describing the details of this method is in preparation (personal communication, Sander van Riet, Leiden University Medical Center, Leiden, the Netherlands). Briefly, tumour-free peripheral lung tissue was collected from patients undergoing cancer resection and subjected to enzymatic digestion. The use of lung tissue that was collected within the framework of patient care was in line with the Federation of Dutch Medical Scientific Societies code of conduct [20]. This code of conduct describes the opt-out system for coded anonymous further use of such samples. Cells were cultured as organoids in Basement Membrane Extract organoid matrix using feeder-free conditions. Organoids retained AT2 cell characteristics as shown by positive staining for proSFTPC and HTII-280 and were used at passage 1 for these experiments.

### Mouse tissue

Tissue from inducible SFTPC-I73T-expressing mice [18] was a kind gift from Michael Beers (University of Pennsylvania, Philadelphia, PA, USA).

Unless stated, figures depict representative images from at least three independent repeats.

## Results

### When compared with wild-type SFTPC, the I73T variant is mislocalised, misprocessed and aberrantly O-glycosylated

When detected using N-terminal domain (Npro) antibodies, we confirmed that wild-type proSFTPC (SFTPC-WT) localises to punctate structures consistent with MVBs [21, 22] and that the SFTPC-I73T variant redistributes to the plasma membrane in AT2 cells of SFTPC-I73T transgenic mouse lung (figure 1b) [10, 18].

To investigate the mechanisms responsible, a cell model of early SFTPC trafficking was established. Primary AT2 cells are unsuitable for detailed mechanistic work due to the complexity of genetic manipulation required and their dedifferentiation when grown in monolayer culture [23]. HeLa cells have long been used to undertake studies of protein trafficking and there already exists a large bioresource of genetically altered HeLa lines with which to interrogate the generic biological processes responsible for anterograde trafficking. Enhanced green fluorescent protein-proSFTPC (GFP-SFTPC) was expressed in HeLa cells where the GFP-tag did not affect localisation compared with untagged proteins (supplementary figure S1a). Surface accumulation of GFP-SFTPC-I73T relative to GFP-SFTPC-WT could be detected by microscopy and flow cytometry using an antibody to the BRICHOS domain, although interestingly we could detect low levels of SFTPC-WT at the plasma membrane (figure 1c). In addition, GFP-SFTPC-I73T accumulated in tubular structures (figure 1c) which MICAL-L1 and Rab8 co-staining demonstrated to be recycling endosomes (supplementary figure S1b).

SFTPC from cell lysates migrated as species of multiple sizes without or with the GFP tag (figure 1d and supplementary figure S1c). GFP-SFTPC-WT migrated as a doublet of ∼50 kDa and a smaller band at ∼34 kDa (arrowhead). The doublet represents full-length proSFTPC±palmitoylation [15, 24]. In keeping with previous studies [10], SFTPC-I73T migrated more slowly and accumulated an additional species “SFTPC*” (*, figure 1d and supplementary figure S1c). Affinity-purified GFP-SFTPC-WT and GFP-SFTPC-I73T were subjected to mass spectrometry, which confirmed the upper bands to be full-length protein, whereas the smallest band lacked the BRICHOS domain and linker, and the SFTPC* band in GFP-SFTPC-I73T samples lacked the BRICHOS domain, but retained the linker (supplementary figure S1d).

To determine the relevance of the I73 residue during SFTPC maturation, a GFP-SFTPC-I73A variant was generated. Like GFP-SFTPC-I73T, this localised to the plasma membrane (supplementary figure S2a) and accumulated an SFTPC*-like species (supplementary figure S2b). However, bands of GFP-SFTPC-I73A migrated with sizes similar to GFP-SFTPC-WT, albeit in proportions similar to those of GFP-SFTPC-I73T. O-glycosylation frequently occurs on threonine residues of membrane proteins during trafficking [25], so we hypothesised that retardation of SFTPC-I73T might reflect O-glycosylation of T73. This was confirmed by mass spectrometry and by observing that O-glycosidase treatment reverted the migration of GFP-SFTPC-I73T to that of GFP-SFTPC-WT (supplementary figure S2c and d).

Mutation of I73 therefore leads to SFTPC accumulation at the cell surface and in recycling endosomes, and promotes accumulation of both uncleaved proSFTPC and the partially processed SFTPC* form, which retains the linker, but lacks the BRICHOS domain. This indicates that I73 plays an important role in SFTPC trafficking and proteolysis of its linker region. The observed O-glycosylation of 73T is not responsible for SFTPC mislocation, but might plausibly have as yet unstudied toxic effects.

### SFTPC-WT traffics to the plasma membrane

**FIGURE 2:**
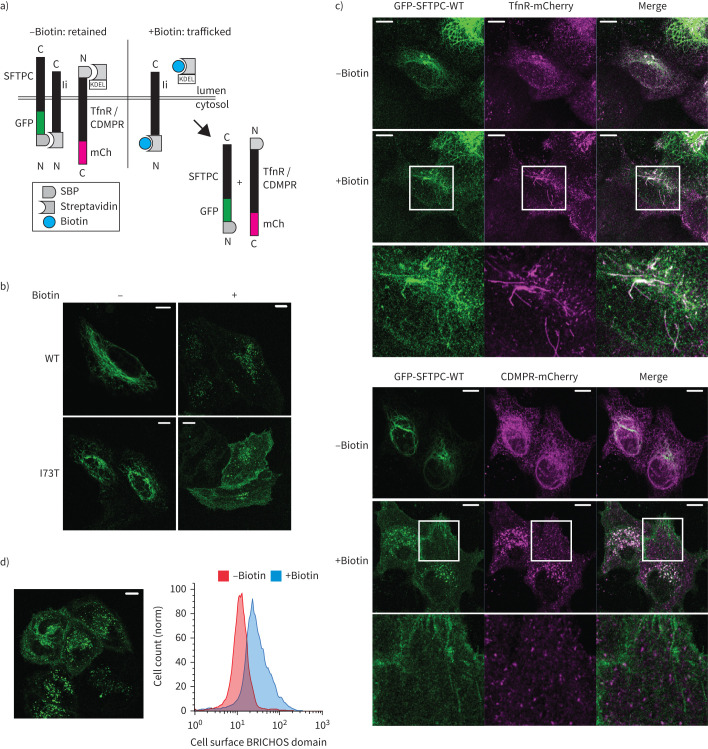
All surfactant protein C (SFTPC) is tracked from early compartments to the plasma membrane. a) Schematic of Retention Using Selective Hooks (RUSH) system, in which proteins are held in early tracking compartments using a streptavidin “hook” before release after addition of biotin. b) Localisation of SFTPC in HeLa cells expressing the RUSH-green fluorescent protein (GFP)-SFTPC fusion protein treated±biotin for 16 h confirms they traffic normally to post-Golgi compartments. c) Real-time tracking of GFP-SFTPC with transferrin receptor (TfnR) or CDMPR reveals co-localisation of SFTPC with TfnR in tubular structures, but failure of co-localisation beyond the Golgi with CDMPR which is seen in vesicles. d) After 2 h of biotin exposure, GFP-SFTPC-wild type (WT) is visible at the plasma membrane; this is reflected in increased BRICHOS domain presence at the plasma membrane by flow cytometry. SBP: streptavidin-binding protein, mCh: mCherry. Scale bars=10 μm.

ProSFTPC is believed to traffic directly from the Golgi to an acidic compartment for proteolysis [26, 27]. For direct Golgi to endosome trafficking, cytosolic adaptors (*e.g.* AP1 or GGA proteins) recognise targeting motifs (*e.g.* YXXØ or (DE)XXX(LL)) to facilitate packaging into clathrin-coated vesicles [28]. The cytosolic domain of SFTPC lacks such sequences, but GGA proteins (especially GGA2) can recognise some ubiquitinated cargoes lacking targeting sequences [29]. Since SFTPC can be ubiquitinated on lysine 6 (K6), potentially allowing recognition by GGAs, we knocked-down GGA1–3, but detected no effect on GFP-SFTPC localisation or proteolysis (supplementary figure S3a and b). However, since gradual depletion of adaptor proteins following knockdown may allow cellular compensation, we next tested the effect of rapid GGA2 inactivation using “knock-sideways” in which GGA2 is relocalised to mitochondria instantaneously upon addition of rapamycin [30]. The Retention Using Selective Hooks (RUSH) system enabled visualisation of SFTPC trafficking in living cells (figure 2a) [31]. Streptavidin-binding protein (SBP)-GFP-SFTPC initially localised to the ER, but upon addition of biotin relocalised in patterns resembling GFP-SFTPC (figure 2b). Following biotin-induced release, SBP-GFP-SFTPC-WT trafficked to distal compartments including the plasma membrane, while no effect of GGA2-knocksideways was observed (supplementary figure S3c and d). This led us to hypothesise that all SFTPC, including the wild type, might traffic *via* the cell surface and not directly from the Golgi to endosomes.

To test this, we compared SFTPC trafficking with that of transferrin receptor (TfnR) and CDMPR, which traffic *via* the plasma membrane or directly to the endolysosomal system, respectively (figure 2a) [32]. Strikingly, SFTPC-WT trafficked *via* tubular post-Golgi structures with TfnR, without co-localisation with CDMPR, which exited the Golgi apparatus in vesicles (figure 2c). As expected, SBP-GFP-SFTPC-I73T also trafficked with TfnR then trafficked to the plasma membrane (supplementary figure S4a and b) [10]. Remarkably, SBP-GFP-SFTPC-WT also trafficked to the plasma membrane, as visualised by imaging and flow cytometry (figure 2d).

These data show that both SFTPC-WT and SFTPC-I73T traffic directly to the plasma membrane from the Golgi. Thus, the accumulation of SFTPC-I73T at the cell surface represents aberrant retention, rather than mistrafficking *per se*.

### SFTPC-WT is retrieved from the plasma membrane by AP2-dependent endocytosis then targeted onwards by K63 oligo-ubiquitination

**FIGURE 3:**
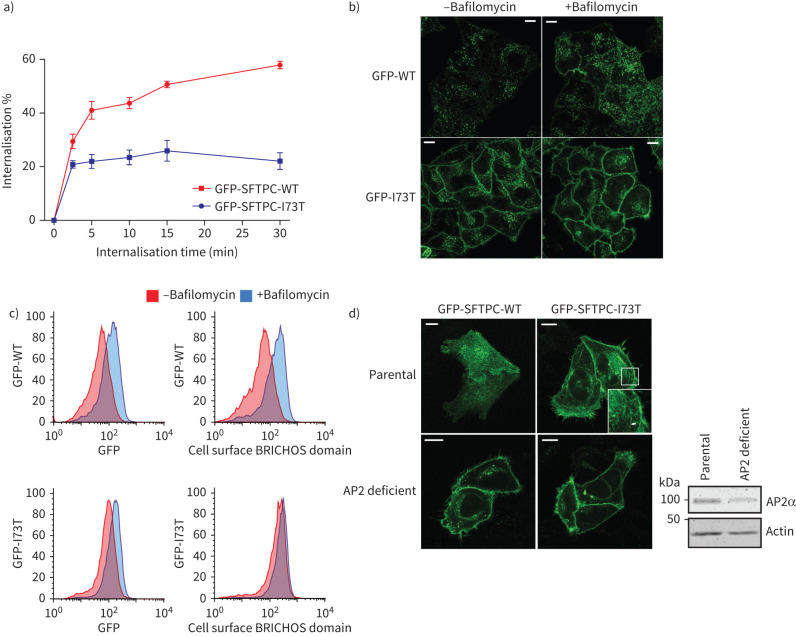
Surfactant protein C (SFTPC) internalisation from the plasma membrane is AP2 dependent and is retarded by the I73T mutation. a) Quantitative SFTPC internalisation assay. Cells expressing green fluorescent protein (GFP)-SFTPC-wild type (WT) or -I73T were labelled with a BRICHOS domain antibody on ice, then protein allowed to internalise at 37°C for the indicated times. Cells were placed back on ice, fixed but not permeabilised, and labelled with a secondary antibody before analysis by flow cytometry. n=3. Data are presented as mean±sem. b) and c) HeLa cells stably expressing GFP-SFTPC-WT or -I73T were treated with 30 µM bafilomycin for 16 h to inhibit clathrin-mediated endocytosis. This results in cell-surface SFTPC accumulation as seen by confocal microscopy and measured by flow cytometry. d) AP2-deficient cells were used to assess localisation of Retention Using Selective Hooks (RUSH)ed GFP-SFTPC-WT and -I73T after 2 h of biotin treatment. AP2M1 CRISPR knockout cells were immunoblotted for the AP2α-subunit. Although some α-subunit remains, this complex is nonfunctional. Scale bars=10 μm.

We next studied the trafficking of SFTPC from the plasma membrane using antibody feeding: cell-surface SFTPC was labelled with primary antibody on ice, incubated at 37°C to allow internalisation, and then remaining cell surface protein detected using a fluorescently conjugated secondary antibody. Labelled GFP-SFTPC-WT was internalised more completely than GFP-SFTPC-I73T, although there was evidence of some early SFTPC-I73T internalisation (figure 3a). This was unaffected by the fluorescent GFP tags introduced to visualise trafficking (supplementary figure S5). The plateauing of the I73T signal may reflect recycling of labelled protein back to the plasma membrane during the course of the assay. The rapidity of initial SFTPC internalisation was consistent with clathrin-mediated endocytosis (CME). Prolonged bafilomycin treatment, which inhibits CME [33], caused accumulation of GFP-SFTPC-WT at the plasma membrane, but had little effect on the distribution of GFP-SFTPC-I73T (figure 3b and c). The involvement of CME was confirmed in AP2-deficient cells, where GFP-SFTPC-WT and GFP-SFTPC-I73T both accumulated at the plasma membrane (figure 3d).

**FIGURE 4:**
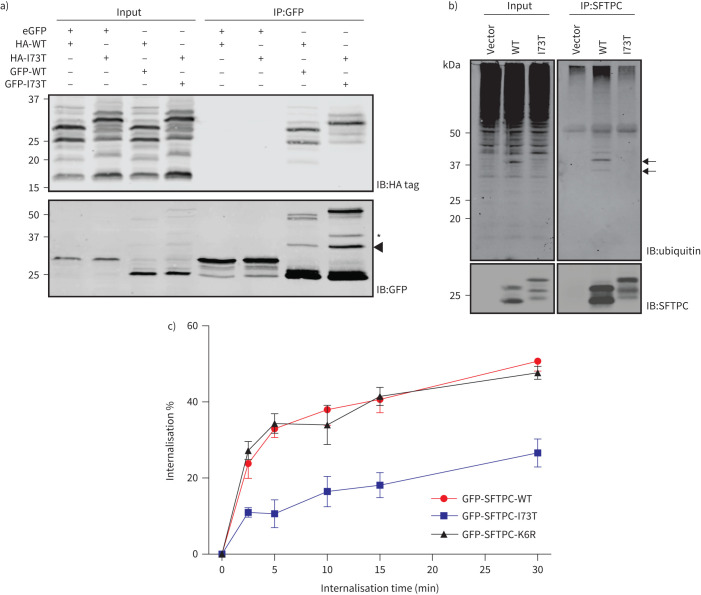
Retardation of surfactant protein C (SFTPC)-I73T internalisation is not due to failed oligomerisation or ubiquitination. a) HeLa cells were transfected with haemagglutinin (HA)-tagged and green fluorescent protein (GFP)-tagged SFTPC (wild type (WT) or I73T) and lysates subjected to anti-GFP immunoprecipitation before immunoblotting for the HA tag and GFP. Both SFTPC-WT and SFTPC-I73T are equally able to oligomerise. b) Lysates from HeLa cells expressing SFTPC-WT or SFTPC-I73T were subjected to SFTPC immunoprecipitation and immunoblotted for ubiquitin. Note the presence of oligoubiquitinated SFTPC-WT (arrows) which is not seen in I73T-expressing cells. c) Quantitative SFTPC internalisation assay of cells expressing GFP-SFTPC-WT, I73T or K6R. n=3. Data are presented as mean±sem.

Therefore, the I73T mutation partially impairs CME despite being topologically unable to interact with sorting factors such as AP2. Many membrane proteins undergo clustering to promote CME. Indeed, oligomerisation of SFTPC is known to occur during its trafficking [21]. However, immunoprecipitation of co-expressed GFP-SFTPC and HA-SFTPC yielded hetero-oligomers of both SFTPC-WT and SFTPC-I73T, suggesting that impaired endocytosis of SFTPC-I73T is unlikely to be explained by failure of oligomerisation (figure 4a).

We next considered the possibility that aberrant ubiquitination of SFTPC-I73T contributes to disrupted trafficking. SFTPC is ubiquitinated on residue K6, which has been reported to be involved in endosomal targeting [13, 14]. Immunoprecipitation of untagged and GFP-tagged SFTPC confirmed oligoubiquitination of SFTPC-WT that was absent in SFTPC-I73T (figure 4b and supplementary figure S6a). We generated GFP-SFTPC-K6R and confirmed this was not ubiquitinated (supplementary figure S6b). However, the rate of SFTPC-K6R internalisation was indistinguishable from that of SFTPC-WT indicating that ubiquitination is unnecessary for endocytosis and therefore cannot account for differing rates of endocytosis between SFTPC-WT and SFTPC-I73T (figure 4c and supplementary figure S5).

**FIGURE 5:**
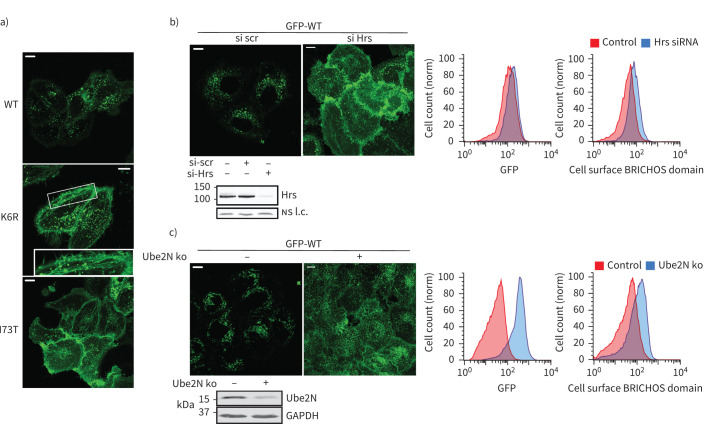
Surfactant protein C (SFTPC) ubiquitination in K63 chains is required for tracking into multivesicular bodies (MVBs). a) Confocal imaging of HeLa cells expressing green fluorescent protein (GFP)-SFTPC-wild type (WT), ubiquitination-deficient K6R and I73T reveals that like I73T, SFTPC-K6R is redistributed to the cell surface and into recycling endosomes (middle panel, inset). b) HeLa cells were transfected with either scrambled (scr) or ESCRT0 protein Hrs siRNA for 48 h. Immunoblotting confirmed knockdown and confocal imaging revealed dramatic redistribution of GFP-SFTPC-WT to the plasma membrane. Flow cytometry con­firmed a slight increase in total GFP plus partial redistribution of SFTPC to the plasma membrane in the presence of Hrs siRNA. c) A Ube2N CRISPR knockout pool was made and success confirmed by immunoblot. Confocal microscopy revealed both an overall increase in GFP signal along with redistribution of GFP-SFTPC-WT to the plasma membrane in these cells, confirmed by flow cytometry. ns: nonsignificant; l.c.: loading control. Scale bars=10 μm.

The observed accumulation of SFTPC-I73T in recycling endosomes suggests failure of sorting at endosomes towards MVBs. By microscopy, we observed that ubiquitination-deficient GFP-SFTPC-K6R localised to the plasma membrane and recycling endosomes (figure 5a and supplementary figure S6c). Since internalisation of proteins into intraluminal vesicles of MVBs involves recognition of ubiquitinated cargo by the endosomal-sorting complex required for transport (ESCRT) machinery [34], we depleted cells of ESCRT0 protein Hrs [35] and observed a dramatic relocalisation of GFP-SFTPC-WT to recycling endosomes and the plasma membrane (figure 5b). K63 chains of ubiquitin generated by the E2-ligase (Ube2N) [36] mark proteins for trafficking to endosomes [34]. When Ube2N was depleted, GFP-SFTPC-WT again redistributed to recycling endosomes and the plasma membrane (figure 5c).

These results demonstrate that both SFTPC-WT and SFTPC-I73T are endocytosed in an AP2-dependent manner, although SFTPC-I73T is internalised less efficiently. K63 ubiquitination of SFTPC-WT then directs it to MVB intraluminal vesicles. Failure of this sorting event redirects SFTPC-I73T to the plasma membrane *via* recycling endosomes.

### SFTPC undergoes C-terminal cleavage following endocytosis prior to entering the MVB

Proteolytic maturation of SFTPC occurs in acidic post-Golgi compartments [26, 37]. A cathepsin H-mediated N-terminal cleavage in MVBs [27] is preceded by C-terminal cleavages at an unknown location. Cleavage events occurring before MVB entry can be studied in HeLa cells, whereas assessment of N-terminal proteolysis in later compartments requires the presence of all SFTPC trafficking compartments (including lamellar bodies) and was therefore not studied further here.

**FIGURE 6:**
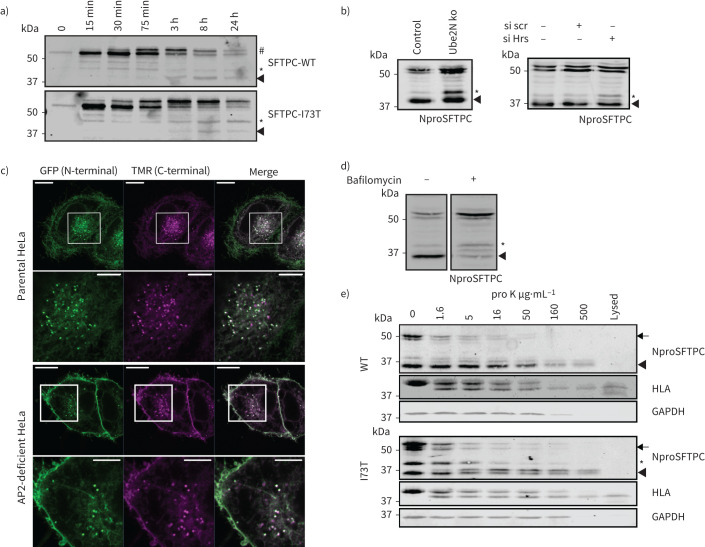
Surfactant protein C (SFTPC) C-terminal cleavage occurs after transit *via* the plasma membrane, but prior to multivesicular body (MVB) internalisation. a) HeLa cells expressing the Retention Using Selective Hooks (RUSH)-SFTPC fusion protein were allowed to traffic for the times indicated before lysates were subjected to green fluorescent protein (GFP) immunoprecipitation and NproSFTPC immunoblotting. The intermediate forms (* and arrowhead) appear contemporaneously for SFTPC-wild type (WT) and -I73T, but the first I73T intermediate (*) accumulates as the WT intermediate is cleared. b) HeLa cells deficient in Ube2N or Hrs in which SFTPC cannot enter MVBs accumulate SFTPC*. c) HeLa cells were transfected with GFP-SFTPC-Halo and the Halotag labelled with TMR ligand for 15 min before fixing. d) Treatment of GFP-SFTPC-WT expressing HeLa cells with 100 nM bafilomycin for 16 h results in preferential accumulation of full-length proprotein. e) HeLa cells stably expressing GFP-SFTPC-WT or I73T were incubated with proteinase K to digest exposed proteins at the plasma membrane before being lysed and subjected to immunoblot for SFTPC, human leukocyte antigen (HLA) and GAPDH. The full-length (WT and I73T) and intermediate (SFTPC*) species are digested by proteinase K, suggesting that they reside at least partially at the cell surface. Lysed: lysis before proteinase K treatment. Scale bars=10 μm or 5 μm (zoomed images).

Using the RUSH system, at 30 min following ER release we observed that full-length SFTPC-WT and SFTPC-I73T both underwent palmitoylation giving rise to a slower migrating band visualised by immunoblot (#; figure 6a). From 3 h, full-length SFTPC-WT was lost while a 37-kDa C-terminally cleaved product appeared (arrowhead). In contrast, more full-length SFTPC-I73T was retained and the SFTPC* product accumulated. A similar band was observed early in SFTPC-WT-expressing cells, but disappeared by 3 h. We conclude that early C-terminal proteolysis generating SFTPC* occurs in a compartment accessible to both SFTPC-WT and SFTPC-I73T. The selective accumulation of SFTPC* with SFTPC-I73T expression suggests that sequential C-terminal processing is impaired by I73T. To determine if this is a cause or consequence of aberrant trafficking, we deleted Ube2N or depleted ESCRT0 protein Hrs to impair MVB import. Both caused WT-SFTPC* to accumulate, suggesting that access to MVBs is necessary for the final C-terminal cleavage (figure 6b). The persistence of low levels of C-terminally cleaved SFTPC in all conditions, suggests an incomplete trafficking block or some redundancy in SFTPC sorting.

To determine the subcellular location of C-terminal proteolysis, we generated GFP-SFTPC-WT-HaloTag and confirmed its correct trafficking and cleavage (supplementary figure S7). The majority of GFP-SFTPC-WT-HaloTag-containing puncta retained both tags, identifying them as compartments prior to C-terminal proteolysis (figure 6c). In AP2-deficient cells, uncleaved GFP-SFTPC-WT-HaloTag accumulated at the plasma membrane, indicating that full-length SFTPC first reaches the cell surface and undergoes C-terminal proteolysis following endocytosis. This finding is further supported by the observation that bafilomycin-mediated inhibition of endocytosis results in accumulation of full length proSFTPC but has a relatively smaller effect on the abundance of the * intermediate (figure 6d).

We sought to determine whether impaired C-terminal proteolysis contributes to mislocalisation of SFTPC-I73T or is a consequence. Flow cytometric detection of cell-surface BRICHOS domain (uncleaved protein) had shown less dramatic differences between SFTPC-WT and SFTPC-I73T than were observed by imaging the cytosolic GFP-tag (figure 1c). Similar results had been obtained when SFTPC-WT was redistributed to the cell surface by impairing endosomal sorting (figure 5b and c). This suggested that recycled SFTPC might undergo C-terminal proteolysis prior to recycling. To investigate this, we treated intact cells with proteinase K to digest surface-exposed proteins (figure 6e). Human leukocyte antigen (HLA) served as a control for surface exposure, while GAPDH reported cytosolic proteolysis. Full-length SFTPC-WT and SFTPC-I73T were digested by low concentrations of proteinase K similar to those required for HLA digestion, confirming their presence at the cell surface. The fully C-terminally cleaved 37-kDa band, by contrast, resisted proteolysis to a similar extent as GAPDH suggesting an intracellular localisation. SFTPC* showed an intermediate sensitivity to proteinase K, consistent with a mixed surface and endosomal localisation. As the proteinase K digestion was performed on ice, no trafficking of SFTPC could occur during this incubation. Consequently, loss of the SFTPC* intermediate reflects its digestion by proteinase K, rather than being an indirect effect of losing full-length proSFTPC.

Full-length SFTPC is therefore first trafficked to the cell surface and then, following AP2-dependent endocytosis, C-terminal proteolysis occurs distal to the linker domain to yield SFTPC*. This is followed by a juxta-membrane cleavage to generate fully C-terminally cleaved SFTPC. While SFTPC-WT is then rapidly transported to the MVB for N-terminal proteolysis, the I73T mutation impairs juxta-membrane cleavage, leading to recycling of SFTPC* to the cell surface.

### SFTPC cleavage is necessary for targeting away from the cell surface to MVBs

**FIGURE 7:**
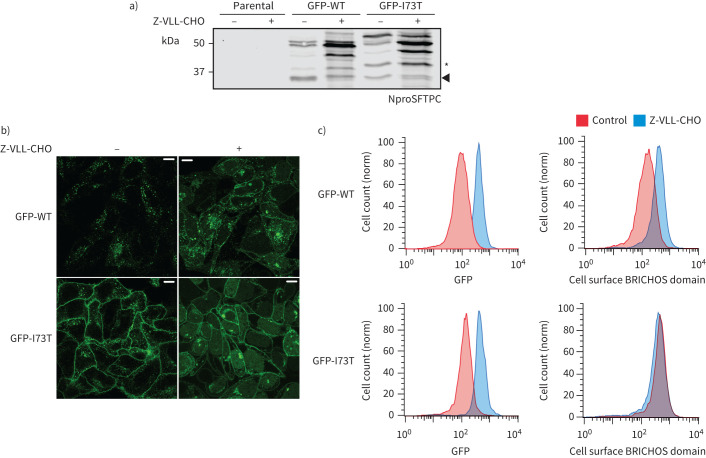
Surfactant protein C (SFTPC) C-terminal cleavage is inhibited by Z-VLL-CHO. a) HeLa cells stably expressing green fluorescent protein (GFP)-SFTPC-wild type (WT) or -I73T were treated with 5 μM Z-VLL-CHO for 16 h and lysates immunoblotted for NproSFTPC. Cells treated with Z-VLL-CHO develop an excess of early C-terminal processing intermediates, increased overall SFTPC (as measured by GFP) and redistribution of SFTPC to the plasma membrane, as seen by b) confocal microscopy and c) flow cytometry. Scale bar=10 μm.

We wished to determine whether proteolytic removal of the linker region serves as a signal to divert SFTPC to MVBs and away from recycling to the plasma membrane. Another BRICHOS-domain-containing protein, Bri2, undergoes sequential cleavage first by furin distant from the membrane and then closer to the membrane by the membrane-bound sheddase ADAM10 [38]. Our attempts to insert tags between the transmembrane domain and linker impaired juxta-membrane cleavage especially with longer tags (supplementary figure S8). This disproportionately affected SFTPC-WT leading to accumulation of an SFTPC*-like product. Suspecting that juxta-membrane cleavage might be mediated by an endosomal membrane-bound sheddase, we treated cells with Z-VLL-CHO, an inhibitor of such proteases. Z-VLL-CHO dramatically increased levels of SFTPC-WT in lysates, predominantly as full-length protein and BRICHOS-cleaved intermediates (figure 7a) with accumulation of SFTPC-WT at the cell surface (figure 7b and c). Proteolytic processing of SFTPC-I73T was further impaired by Z-VLL-CHO, and SFTPC-I73T remained at the cell surface.

**FIGURE 8:**
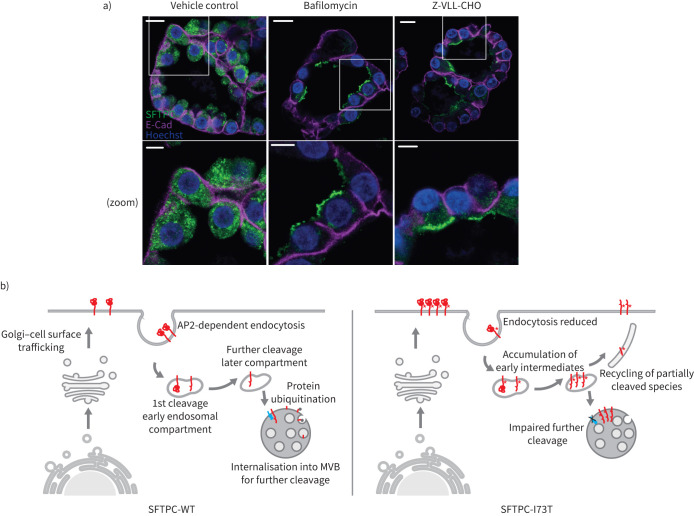
Surfactant protein C (SFTPC) is tracked *via* the cell surface in human alveolar organoids. a) Human alveolar type 2 (AT2) cell organoids were treated with bafilomycin or Z-VLL-CHO and the localisation of SFTPC determined by immunohistochemistry. E-cadherin (E-Cad) was used to delineate the basolateral membranes. Note that both compounds redistribute SFTPC to the apical plasma membrane. Scale bars=10 μm or 5 μm (zoomed images). b) Proposed model of SFTPC tracking. SFTPC pro-protein is tracked to the plasma membrane from the Golgi apparatus before AP2-dependent endocytosis in clathrin-coated vesicles. Initial C-terminal cleavage occurs in an early endocytic compartment before further cleavage in a later compartment by a membrane protease. This facilitates ubiquitination and allows internalisation into multivesicular bodies (MVBs) for onward cleavage and packaging into lamellar bodies. In the presence of the I73T mutation, endocytosis is reduced, early intermediates accumulate and are recycled due to a later block in tracking mediated by failure of later cleavage, ubiquitination and MVB internalisation.

Since SFTPC is expressed exclusively in AT2 cells, it was necessary to validate these findings in a physiologically relevant model. We therefore generated organoids from primary AT2 cells isolated from human lungs. These lung organoids were composed of polarised AT2 cells with apical membranes directed towards the lumen. Immunofluorescence demonstrated endogenous SFTPC-WT in punctate structures beneath the apical surface, while treatment with either bafilomycin (to inhibit CME) or Z-VLL-CHO (to inhibit endosomal sheddases) redistributed endogenous proSFTPC into AT2 apical membranes (figure 8a). These findings align with the trafficking route mechanistically explored in HeLa cells.

Taken together, these experiments reveal that SFTPC undergoes proteolysis distal to the linker domain in endosomes followed by juxta-membrane cleavage, likely by a membrane-bound sheddase. Impaired juxta-membrane cleavage, either induced pharmacologically or by the I73T mutation, results in unproductive recycling of SFTPC between endosomes and the plasma membrane (figure 8b).

## Discussion

We set out to understand the mechanisms by which SFTPC-I73T mistraffics to provide insight into its role in FPF. In doing so, we have defined the itinerary of wild-type SFTPC, which involves trafficking *via* the plasma membrane. We observed that residue I73 of SFTPC, located within the extracellular linker region of the proprotein, is key to trafficking as its mutation results in impaired progression to MVBs and unproductive SFTPC recycling between the cell surface and early endosomes.

Aberrant retention of the linker region is associated with failure of SFTPC ubiquitination, which does not affect endocytosis, but impairs subsequent internalisation of SFTPC into MVBs. Our work delineates a mechanism that explains the previous observation that SFTPC-I73T localises to early endosomes while wild-type protein is found in MVBs [6]. The precise mechanism through which linker cleavage results in MVB targeting of wild-type SFTPC remains unclear, but linker proteolysis may be necessary to facilitate sorting into late endosomes and/or enables K63-ubiquitination. The partial block of trafficking of SFTPC-I73T seen both in cultured cells and in SFTPC-I73T transgenic mice [18] suggests that I73T produces an incomplete block on trafficking and maturation of the protein.

SFTPC is a highly hydrophobic protein thought to promote the spreading of pulmonary surfactant through interaction with phospholipid layers [17]. How SFTPC mutations cause FPF is unclear. Mice lacking SFTPC are viable and grow normally [39], while conditional expression of pathogenic SFTPC mutants results in pulmonary fibrosis [18, 40]. This suggests that the pathogenic mechanism is one of toxic gain-of-function mediated by an abnormal protein rather than loss of function due to SFTPC deficiency. While mutations of the BRICHOS domain causes ER stress [41, 42], the toxic mechanism of linker region mutants is unclear.

Here, we demonstrate that SFTPC-I73T accumulates at the plasma membrane through abnormal recycling from endosomes and from impaired internalisation. Recycled SFTPC-I73T includes the partially cleaved intermediate SFTPC*, consistent with previous plasma membrane profiling data [19]. Interestingly, such abnormal C-terminal SFTPC fragments are present in the bronchoalveolar lavage fluid of individuals with the SFTPC-I73T mutation [6, 43]. Those observations, together with our findings, collectively suggest that C-terminal cleavage products are released into the extracellular space during aberrant recycling of SFTPC. It is tempting to speculate that trafficking of SFTPC-WT *via* the cell surface may have a role in normal AT2 cell function that is perturbed by the I73T mutant. Ongoing work will determine if surface accumulation of SFTPC in its multiple forms, retention in recycling endosomes and/or the released C-terminal cleavage products themselves affect signalling within the AT2 cell or with its neighbours. Understanding the intracellular interactome of SFTPC and its processing intermediates may shed fresh light on this. Alternatively, saturation of protein recycling pathways by SFTPC might have deleterious effects on surface expression of otherwise unrelated proteins.

In this work, by studying a naturally occurring pathogenic variant of SFTPC associated with FPF, we have discovered that all wild-type protein traffics *via* the cell surface to MVBs, rather than directly from the Golgi. The accumulation of the SFTPC-I73T variant at the plasma membrane therefore reflects retardation along its normal route rather than mistrafficking *per se*. Whether SFTPC possesses additional functions that require it to transit *via* the cell surface remains to be established, and may prove crucial in understanding the role of SFTPC-I73T in alveolar dysfunction resulting in FPF. Such insight may also help to clarify the role of alveolar dysfunction in other, nonfamilial forms of IPF.

## Supplementary material

10.1183/13993003.00267-2021.Supp1 **Please note:** supplementary material is not edited by the Editorial Office, and is uploaded as it has been supplied by the author.
Supplementary materials and methods ERJ-00267-2021.Supplement
Supplementary figure S1 ERJ-00267-2021.Figure_S1
Supplementary figure S2 ERJ-00267-2021.Figure_S2
Supplementary figure S3 ERJ-00267-2021.Figure_S3
Supplementary figure S4 ERJ-00267-2021.Figure_S4
Supplementary figure S5 ERJ-00267-2021.Figure_S5
Supplementary figure S6 ERJ-00267-2021.Figure_S6
Supplementary figure S7 ERJ-00267-2021.Figure_S7
Supplementary figure S8 ERJ-00267-2021.Figure_S8

## Shareable PDF

10.1183/13993003.00267-2021.Shareable1 This one-page PDF can be shared freely online.
Shareable PDF ERJ-00267-2021.Shareable
