# Supplementary material for: Novel insights into surfactant protein C trafficking revealed through the study of a pathogenic mutant
Source: Eur Respir J. 2022 Jan 27;59(1):2100267. doi: 10.1183/13993003.00267-2021 (PMC8792467; doi:10.1183/13993003.00267-2021)
Supplement: Supplementary file 1 [file ERJ-00267-2021.Supplement.pdf]

Supplementary Materials and Methods

Chemical reagents

The following chemicals were used: Bafilomycin (Sigma B1973) – 100µM stock solution in DMSO, working concentration 100nM; Z-VLL-CHO (Abcam Ab146640) - 10mM stock solution in DMSO, working concentration 5µM; proteinase K (Sigma p7850).

Antibodies

The following commercial antibodies were used: Npro SFTPC (Abcam Ab90716 / Santa Cruz SC-7706); BRICHOS domain SFTPC (Abgent AP13684b); HA tag (Cell Signalling SC3724); GFP (Abcam Ab1218); Ubiquitin (Cayman Chemical FK2); transferrin receptor (TfnR) (BD bioscience BD-555534); cation-dependent mannose -6-phosphate receptor (CDMPR) (Ab2733); MICAL-L1 (Novus H00085377); GGA3 (BD Bioscience BD612310); AP2 (Novus NB300-721); Ube2N (Abcam Ab109286); HLA (Abcam Ab134189); actin (Abcam Ab3280); GAPDH (Cell signalling 2118 / Merk Millipore MAB374); HTII-280 (Terrace Biotech TB-27AHT2-280); E-cadherin (BD Bioscience BD610182). The Hrs, GGA1 and GGA2 antibodies were kind gifts from the Robinson lab (Cambridge, UK) [1]. The following secondary antibodies were used: IRdye 680/800CW anti-rabbit/mouse 680/800 (LiCor); donkey/ goat anti mouse/ rabbit/ goat AF405/488/594/647 conjugated antibodies (Invitrogen).

Expression vectors

pcDNA-SFTPC, pcDNA-HA-SFTPC and peGFP-SFTPC WT/I73T expression vectors were kind gifts from Professor Mike Beers [2]. The K6R and I73A variants were derived by site directed mutagenesis. HaloTag®-SFTPC, peGFP-SFTPC-linker-HaloTag and internally tagged SFTPC constructs were made either by Gibson assembly or by PCR using oligonucleotides incorporating tag sequences. The Str-KDEL_TfnR-SBP-mCherry/CD-MPR-SBP-mCherry plasmids were a kind gift from David Gershlick (Cambridge, UK) [3]. The Str-Ii_SBP-eGFP-SFTPC constructs were made by Gibson assembly using the Str-Ii_SBP-eGFP-Golgin84 starting plasmid (Addgene 65303). Vectors for CRISPR/Cas9 gene editing were generated using the Zhang protocol[4]. A suitable Ube2N guide sequence was selected from the Brunello library [5] (CCTCAAAGGGGGAATCCTGA) and cloned into the pSpCas9(BB)-2A-mCherry vector which was a kind gift from the Ron lab (Cambridge, UK) [6].

Cell lines and siRNA

mStrawberry-Rab8 HeLa cells, originally made by Andrew Peden (Sheffield, UK) were a kind gift from Matthew Seaman (Cambridge, UK) [7]; The AP2 knockout HeLa pool were a gift from the Bonifacino lab (NIH, USA) [3] and GGA2 knock-sideways HeLa cells a gift from the Robinson lab (Cambridge, UK) [8]. siRNAs (Dharmacon) were introduced by reverse transfection using RNAiMAX (Thermofisher) for 24 hours before refreshing the media: Hrs (UUUACCUCCACUUGUCUCUdTdT); GGA1 (J-013694-08; CACAGGAGUGGGAGGCGAU); GGA2 (J-012908-11; UGAAUUAUGUUUCGCAGAA); GGA3 (J-012881-11; UGUGACAGCCUACGAUAAA). Control siRNA was acquired from Ambion (Texas, USA).

Western blotting and immunofluorescence

Triton lysis, SDS-PAGE electrophoresis followed by immunoblotting and immunofluorescence of immortalised cells were performed as previously described [9] [10].

Paraffin embedded tissue was deparaffinised and rehydrated using xylene (3x5min) and EtOH (2 minutes each of 100%, 70%, 50%, 0%) then antigen retrieval undertaken by boiling in 10mM sodium

citrate, 0.05% tween20 pH 6.0 for 10 minutes. Samples were blocked with 1% BSA then incubated overnight with primary antibody at 4°C then secondary antibody diluted 1:500 for 1 hour at room temperature. Hoechst (Invitrogen) 1:5000 was added to the penultimate wash before mounting with Prolong Gold Antifade (Thermofisher).

Blots were developed using a LiCor Odyssey and all imaging undertaken on an LSM880 confocal microscope.

Immunoprecipitation

Briefly, for SFTPC immunoprecipitation 30µl Sepharose A bead slurry (Lifetech 10-1041) were bound to a 2µl non-specific Rb IgG (Ab37415) or 1µg SFTPC Ab (Ab90716) per sample for 1 hour at 4°C. Cells were lysed in 1% Triton lysis buffer before normalised amounts of protein were precleared then transferred to the IP beads and incubated overnight at 4°C. Protein was eluted into SDS loading buffer. GFP-Trap®-magnetic agarose beads (Chromotek) were used following the manufacturer's protocol and the recommended 0.5% NP-40 lysis buffer. Samples were incubated with beads for 1 hour before washing in RIPA buffer to minimise non-specific binding.

Proteinase K assay

HeLa cells stably expressing GFP-SFTPC-WT or GFP-SFTPC-I73T were resuspended in PBS with calcium/magnesium. Proteinase K (Sigma p7850) was added on ice for 1 hour then inhibited with 5mM PMSF before proceeding to cell lysis.

O-Glycosylation assay

Lysates from cells stably expressing GFP-SFTPC-WT or GFP-SFTPC-I73T were heated with 1x New England Biolabs (NEB) glycoprotein denaturing buffer to 100°C for 10 minutes the treated with neuraminidase (NEB 0720) or O-glycosidase (NEB 0733) at 37°C for 4 hours in appropriate NEB buffers.

Flow cytometry

Cells were detached using 10mM EDTA, washed with cold PBS, blocked with 10% FBS for 30 minutes then incubated with the relevant primary antibody (30 minutes at 4°C) and AF647-conjugated secondary (30 minutes at 4°C). Cells were fixed with 4% PFA then analysed with a BD FACSCalibur and FlowJo, normalising results to the mode. For the quantitative antibody feeding assay, cells were detached using EDTA and resuspended in ice cold media for labelling with primary antibody on ice for 30 minutes. Cells were pelleted and washed with ice cold PBS before being aliquoted and resuspended in cold media. Cold media was replaced with warmed media at the point of returning samples to the incubator for the indicated time periods. At the end of internalisation cells were placed back on ice, washed with cold PBS and incubated with AF647-conjugated secondary antibody at 4°C for 30minutes. Cells were washed again with cold PBS then fixed with 4% PFA and analysed.

Live cell assays and imaging

Imaging was undertaken on an LSM880 confocal microscope or Zeiss Airyscan using Zen black software.

RUSH imaging: Cells were transfected with 2.5µg RUSH vectors 48 hours before imaging. 585µM biotin was added to warm tissue culture media and used to replace the media. For real-time video imaging, a 2x biotin stock was prepared in warm culture media which was added to cells *in situ* before imaging.

Knocksideways assays: GGA2-mito HeLa cells were transfected with 10nM siRNA GGA1-3, incubated for 24 hours then transfected with 3µg SBP-GFP-SFTPC RUSH vectors. 48 hours after

transfection, cells were treated ± 200nM rapamycin to knock GGA2 sideways, then treated ± 585µM biotin for 2 hours before fixing and imaging.

HaloTag labelling: Cells expressing HaloTag fusion proteins were labelled using TMR ligand (Promega) diluted 1:5000 for 10 minutes, washed x3 with media, incubated for 30minutes then fixed and imaged.

Antibody feeding was undertaken by incubating live cells grown on coverslips with the relevant primary antibody diluted in tissue culture media on ice, typically for 30 minutes followed by washing with ice cold PBS. For microscopy, labelled cells were transferred to 37°C for the indicated times before being placed back on ice, then fixed with 4% PFA but not permeabilised. Remaining cell surface protein was labelled with an AF405-conjugated secondary before permeabilising and blocking with 0.1% Triton-X + 10% FBS. An AF594-conjugated antibody was then used to label internalised protein before being washed, stained with DAPI (1:1000 in PBS) and mounted.

Mass spectrometry

Lysates from GFP-SFTPC-expressing HeLas were subjected to anti-GFP immunoprecipitation and eluates separated by SDS-PAGE electrophoresis. Bands were excised, subjected to trypsin digest and run on a Q Exactive Plus spectrometer. Data were processed using PEAKS Studio (version 8.0, Bioinformatics Solutions Inc.) and PTM coverage images exported from the results of the PEAKS Spider stage. A custom R script plotted the coverage of each residue as a percentage of total protein coverage, weighted taking into account the relative abundance (area under the curve) for each peptide. The horizontal dashed line (fig s1C) represents predicted even coverage.

References

1. Hirst J, Lui WW, Bright NA, Totty N, Seaman MN, Robinson MS. A family of proteins with gamma-adaptin and VHS domains that facilitate trafficking between the trans-Golgi network and the vacuole/lysosome. *J Cell Biol* 2000: 149(1): 67-80.
2. Beers MF, Hawkins A, Maguire JA, Kotorashvili A, Zhao M, Newitt JL, Ding W, Russo S, Guttentag S, Gonzales L, Mulugeta S. A nonaggregating surfactant protein C mutant is misdirected to early endosomes and disrupts phospholipid recycling. *Traffic* 2011: 12(9): 1196-1210.
3. Chen Y, Gershlick DC, Park SY, Bonifacino JS. Segregation in the Golgi complex precedes export of endolysosomal proteins in distinct transport carriers. *J Cell Biol* 2017: 216(12): 4141-4151.
4. Ran FA, Hsu PD, Wright J, Agarwala V, Scott DA, Zhang F. Genome engineering using the CRISPR-Cas9 system. *Nat Protoc* 2013: 8(11): 2281-2308.
5. Doench JG, Fusi N, Sullender M, Hegde M, Vaimberg EW, Donovan KF, Smith I, Tothova Z, Wilen C, Orchard R, Virgin HW, Listgarten J, Root DE. Optimized sgRNA design to maximize activity and minimize off-target effects of CRISPR-Cas9. *Nat Biotechnol* 2016: 34(2): 184-191.
6. Amin-Wetzel N, Saunders RA, Kamphuis MJ, Rato C, Preissler S, Harding HP, Ron D. A J-Protein Co-chaperone Recruits BiP to Monomerize IRE1 and Repress the Unfolded Protein Response. *Cell* 2017: 171(7): 1625-1637 e1613.
7. Breusegem SY, Seaman MNJ. Genome-wide RNAi screen reveals a role for multipass membrane proteins in endosome-to-golgi retrieval. *Cell Rep* 2014: 9(5): 1931-1945.
8. Hirst J, Borner GH, Antrobus R, Peden AA, Hodson NA, Sahlender DA, Robinson MS. Distinct and overlapping roles for AP-1 and GGAs revealed by the "knocksideways" system. *Curr Biol* 2012: 22(18): 1711-1716.
9. Malzer E, Szajewska-Skuta M, Dalton LE, Thomas SE, Hu N, Skaer H, Lomas DA, Crowther DC, Marciniak SJ. Coordinate regulation of eIF2alpha phosphorylation by PPP1R15 and GCN2 is required during Drosophila development. *J Cell Sci* 2013: 126(Pt 6): 1406-1415.
10. Dickens JA, Ordonez A, Chambers JE, Beckett AJ, Patel V, Malzer E, Dominicus CS, Bradley J, Peden AA, Prior IA, Lomas DA, Marciniak SJ. The endoplasmic reticulum remains

functionally connected by vesicular transport after its fragmentation in cells expressing Z-alpha1-antitrypsin. *FASEB J* 2016: 30(12): 4083-4097.
