# Supplementary material for: Novel insights into surfactant protein C trafficking revealed through the study of a pathogenic mutant
Source: Eur Respir J. 2022 Jan 27;59(1):2100267. doi: 10.1183/13993003.00267-2021 (PMC8792467; doi:10.1183/13993003.00267-2021)
Supplement: Supplementary file 2 [file ERJ-00267-2021.Figure_S1.pdf]

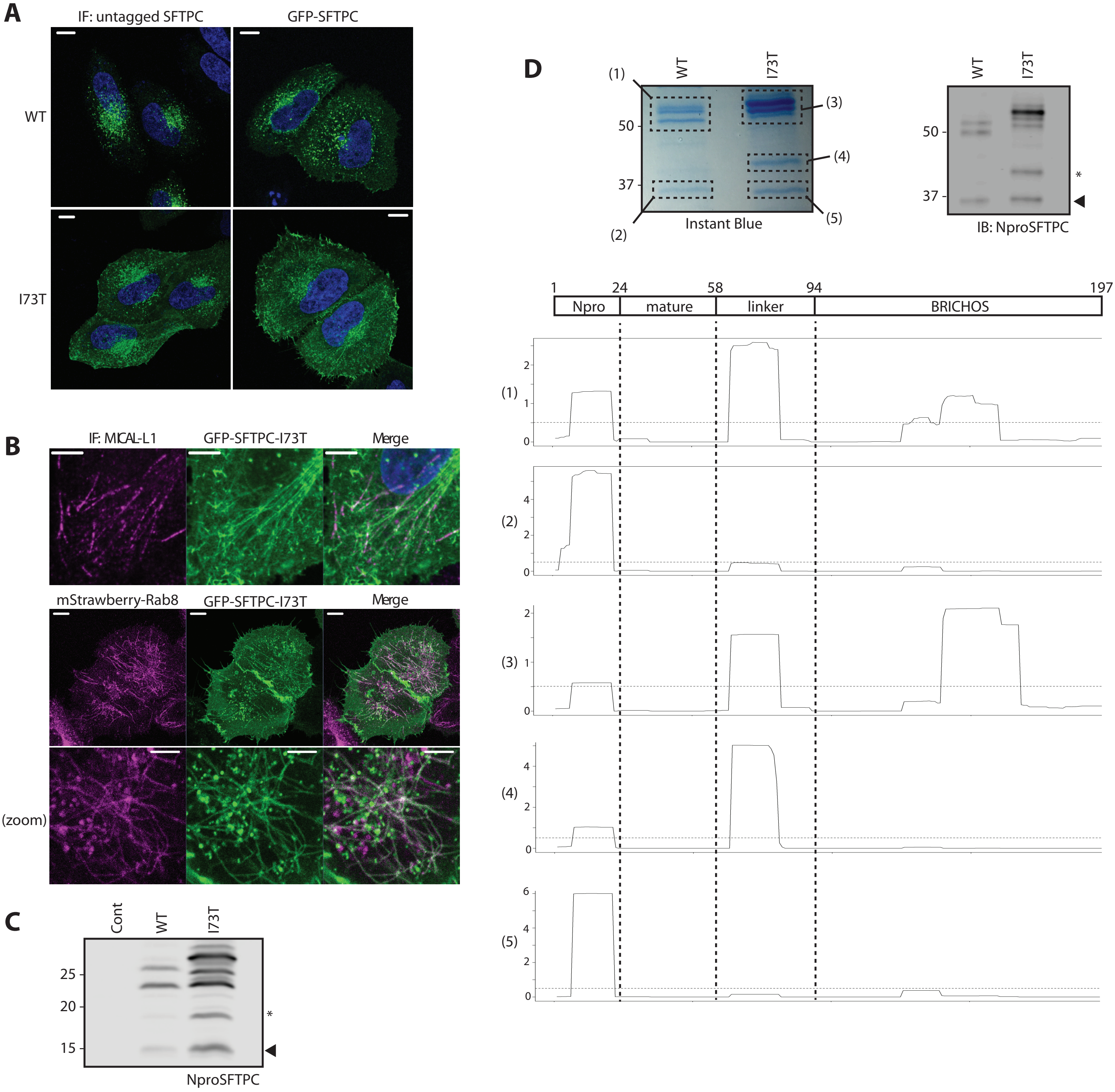

Suppl fig 1. Characterisation of SFTPC expression and cleavage in an immortalised cell system. (A) Confocal imaging of immunostained untagged SFTPC and GFP-SFTPC localisation in HeLa cells. Addition of the GFP tag did not alter the localisation of SFTPC-WT and I73T. (B) Intracellular tubular structures in cells expressing SFTPC-I73T are confirmed as recycling endosomes due to co-localisation with MICAL-L1 (upper panels) and mStrawberry-Rab8 (lower panels). (C) Untagged SFTPC or vector control were transiently expressed in HeLa cells and lysates immunoblotted using an N-terminal SFTPC antibody. (D) Mass spectrometry analysis confirms the aberrant intermediate seen for SFTPC-I73T is partially cleaved but retains the linker region. Pooled lysates from HeLa cells stably expressing GFP-SFTPC-WT and I73T were subjected to anti-GFP immunoprecipitation. Eluates were separated by SDS-PAGE (upper L panel) and relevant bands confirmed by immunoblot (upper R panel). Fragments obtained by trypsin digest were subjected to mass spectrometry. Good coverage was obtained except for the very hydrophobic transmembrane domain. Data displayed as coverage of each residue as a percentage of total protein coverage. Scale bar = 10µm / 5µm (zoomed images).
