# Supplementary material for: Novel insights into surfactant protein C trafficking revealed through the study of a pathogenic mutant
Source: Eur Respir J. 2022 Jan 27;59(1):2100267. doi: 10.1183/13993003.00267-2021 (PMC8792467; doi:10.1183/13993003.00267-2021)
Supplement: Supplementary file 3 [file ERJ-00267-2021.Figure_S2.pdf]

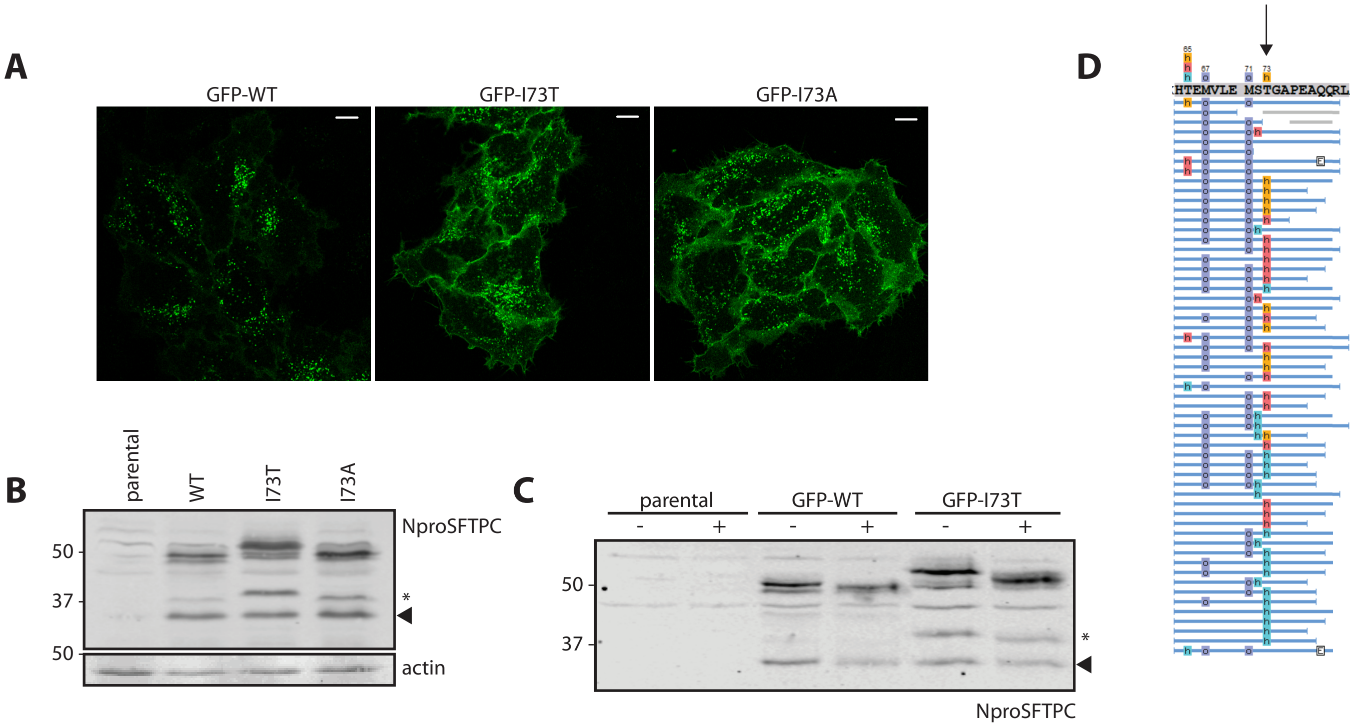

Suppl fig 2. SFTPC I73T is O-glycosylated on T73 but it is the loss of I73, not gain of T73, that causes aberrant trafficking. GFP-SFTPC WT, I73T and I73A were transiently expressed in HeLa cells. Their subcellular localisation was assessed by confocal microscopy (A) and cleavage pattern by immunoblot (B). Note that the loss of I73 determines the mistrafficking phenotype but it is the gain of T73 that alters the band size of full length and early processing intermediates. (C) Immunoblots of HeLa cell lysates following treatment with O-glycosidase revert the band pattern of GFP-SFTPC-I73T to that more similar to GFP-SFTPC-WT. (D) Lysates from HeLa cells expressing GFP-SFTPC-I73T were subjected to GFP-trap immunoprecipitation, separated by protein gel electrophoresis and GFP-SFTPC-I73T bands extracted and subjected to mass spectrometry analysis. Resulting fragments confirm O-glycosylation of T73 (“h” annotation). Scale bar = 10µm.
