# Supplementary material for: Novel insights into surfactant protein C trafficking revealed through the study of a pathogenic mutant
Source: Eur Respir J. 2022 Jan 27;59(1):2100267. doi: 10.1183/13993003.00267-2021 (PMC8792467; doi:10.1183/13993003.00267-2021)
Supplement: Supplementary file 4 [file ERJ-00267-2021.Figure_S3.pdf]

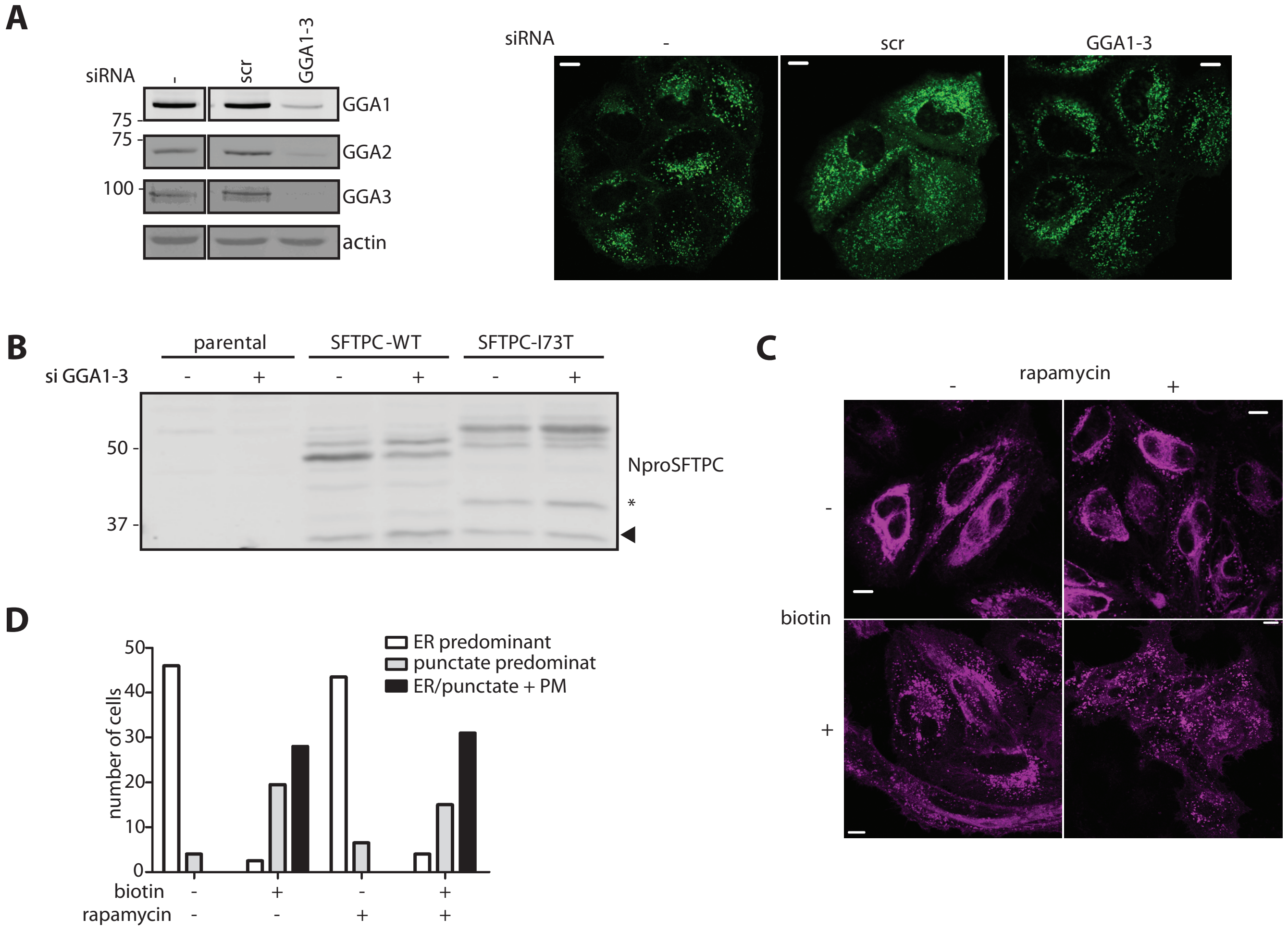

Suppl fig 3. Depletion of GGA proteins does not affect SFTPC trafficking. (A) siRNAs were used to deplete GFP-SFTPC-WT-expressing cells of GGA proteins 1-3, confirmed by immunoblot. The subcellular localisation of SFTPC-WT does not change with knockdown, and nor does the post translational cleavage as measured by immunoblot (B). (C) To mitigate for cellular adaptation to GGA knockdown, a knock sideways system was used. GGA2 was knocked down in GGA2 knocksideways HeLa cells before transfection with GFP-SFTPC-WT RUSH vectors. SFTPC was allowed to traffic by addition of biotin in the presence or absence of GGA2 knocksideways. GGA2 knocksideways does not change SFTPC distribution. Quantification in (D). PM = plasma membrane. Scale bar = 10μm.
