# Supplementary material for: Novel insights into surfactant protein C trafficking revealed through the study of a pathogenic mutant
Source: Eur Respir J. 2022 Jan 27;59(1):2100267. doi: 10.1183/13993003.00267-2021 (PMC8792467; doi:10.1183/13993003.00267-2021)
Supplement: Supplementary file 5 [file ERJ-00267-2021.Figure_S4.pdf]

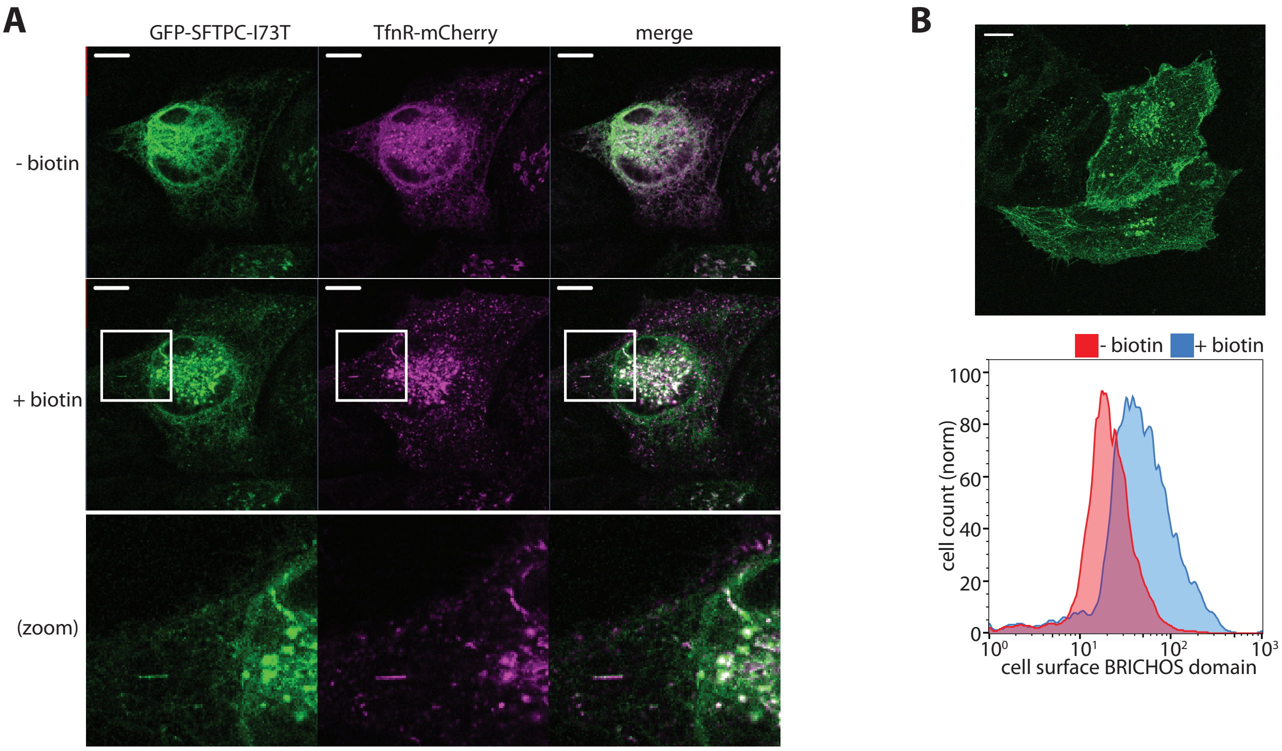

Suppl fig 4. SFTPC-I73T, like SFTPC-WT is trafficked to the plasma membrane from early compartments. (A) GFP-SFTPC-I73T RUSH vectors were co-expressed with TfnR-mCherry and treated with biotin before imaging in real time. Like SFTPC-WT, GFP-SFTPC-I73T traffics with TfnR in tubular structures. (B) After 2 hours of biotin exposure, GFP-SFTPC-I73T is visible at the plasma membrane; this is reflected in increased BRICHOS domain presence at the plasma membrane by flow cytometry. Scale bar = 10µm.
