# Supplementary material for: Novel insights into surfactant protein C trafficking revealed through the study of a pathogenic mutant
Source: Eur Respir J. 2022 Jan 27;59(1):2100267. doi: 10.1183/13993003.00267-2021 (PMC8792467; doi:10.1183/13993003.00267-2021)
Supplement: Supplementary file 6 [file ERJ-00267-2021.Figure_S5.pdf]

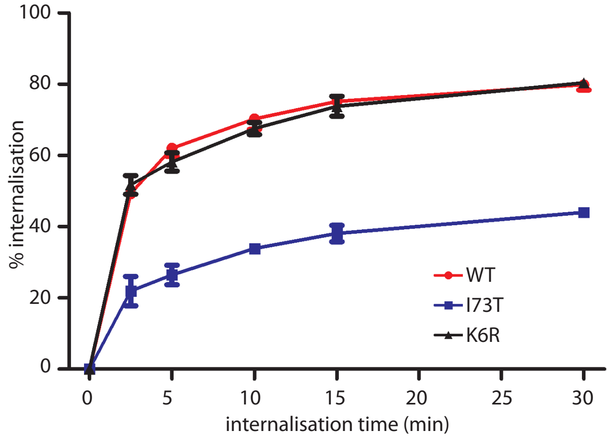

Suppl fig 5. Addition of a GFP tag does not affect SFTPC endocytosis. Quantitative SFTPC internalisation assay. Cells expressing untagged SFTPC-WT, I73T or K6R were labelled with BRICHOS domain antibody on ice, then protein allowed to internalise at 37°C for the indicated times. Cells were placed back on ice, fixed but not permeabilised, and labelled with a secondary antibody before analysis by flow cytometry. n=3, mean +/- sem.
