# Supplementary material for: Novel insights into surfactant protein C trafficking revealed through the study of a pathogenic mutant
Source: Eur Respir J. 2022 Jan 27;59(1):2100267. doi: 10.1183/13993003.00267-2021 (PMC8792467; doi:10.1183/13993003.00267-2021)
Supplement: Supplementary file 7 [file ERJ-00267-2021.Figure_S6.pdf]

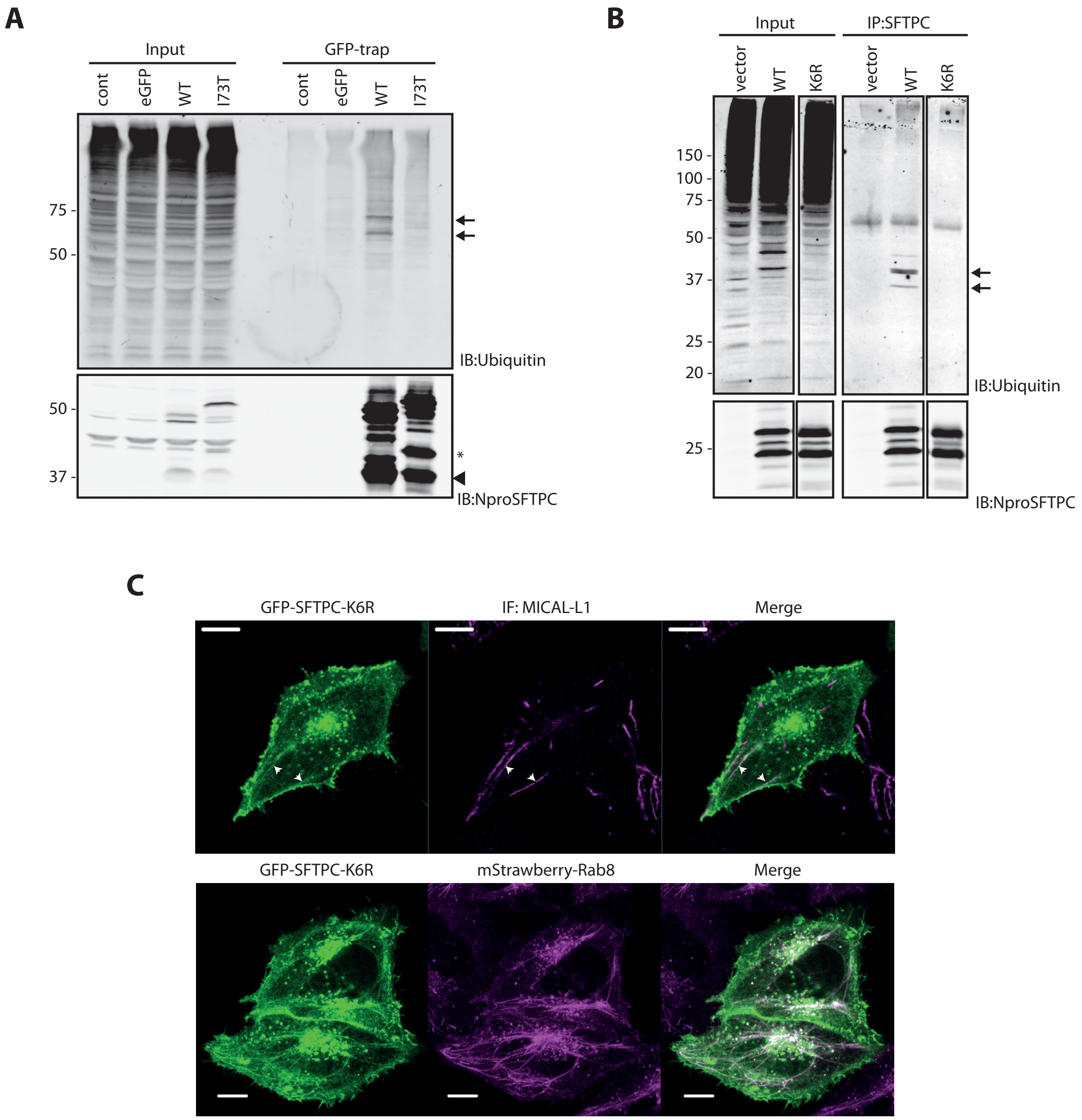

Suppl fig 6. GFP-SFTPC-I73T and K6R fail to be ubiquitinated. (A) Lysates from HeLa cells transfected with vector control, eGFP or GFP-SFTPC were subjected to GFP immunoprecipitation and immunoblotted for ubiquitin and SFTPC. Note the presence of oligoubiquitinated SFTPC-WT (arrows) which is not seen in I73T-expressing cells. (B) Lysates from HeLa cells transfected with vector control, SFTPC-WT or ubiquitination site mutant K6R were subjected to SFTPC immunoprecipitation and immunoblotted for ubiquitin and SFTPC. Note the presence of oligoubiquitinated SFTPC-WT (arrows) which is not seen in the presence of the K6R mutation. (C) Intracellular tubular structures in cells expressing SFTPC-K6R are confirmed as recycling endosomes due to co-localisation with MICAL-L1 (upper panels) and mStrawberry-Rab8 (lower panels). Scale bar = 10μm.
