# Supplementary material for: Novel insights into surfactant protein C trafficking revealed through the study of a pathogenic mutant
Source: Eur Respir J. 2022 Jan 27;59(1):2100267. doi: 10.1183/13993003.00267-2021 (PMC8792467; doi:10.1183/13993003.00267-2021)
Supplement: Supplementary file 8 [file ERJ-00267-2021.Figure_S7.pdf]

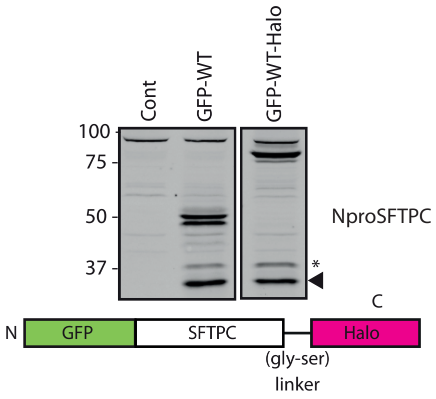

Suppl fig 7. Addition of a C-terminal Halotag does not affect SFTPC cleavage. HeLa cells were transfected with a vector control, GFP-SFTPC or GFP-SFTPC-Halo and lysates immunoblotted for SFTPC. This confirmed that addition of the C-terminal Halotag does not affect post translational cleavage.
