# Supplementary material for: Novel insights into surfactant protein C trafficking revealed through the study of a pathogenic mutant
Source: Eur Respir J. 2022 Jan 27;59(1):2100267. doi: 10.1183/13993003.00267-2021 (PMC8792467; doi:10.1183/13993003.00267-2021)
Supplement: Supplementary file 9 [file ERJ-00267-2021.Figure_S8.pdf]

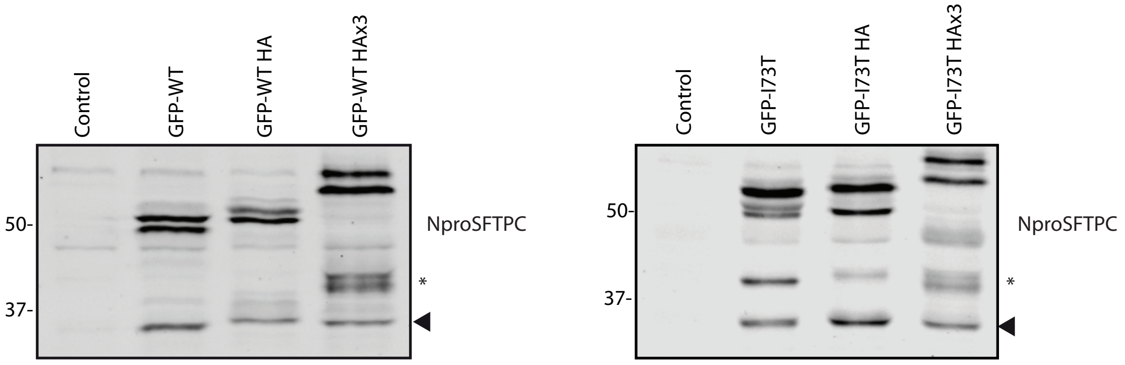

Suppl fig 8. Alteration of the proximal linker region's length with an internal tag affects cleavage. A vector control, one or three HA tags were introduced into the juxta-membrane linker region of GFP-SFTPC (L panel) or I73T (R panel). Expression of these constructs resulted in accumulation of a processing intermediate (*), more marked for SFPTC-WT, consistent with impaired cleavage at the membrane.
