# Supplementary material for: Novel insights into surfactant protein C trafficking revealed through the study of a pathogenic mutant
Source: Eur Respir J. 2022 Jan 27;59(1):2100267. doi: 10.1183/13993003.00267-2021 (PMC8792467; doi:10.1183/13993003.00267-2021)
Supplement: Supplementary file 10 [file ERJ-00267-2021.Shareable.pdf]

SHAREABLE PDF

Novel insights into surfactant protein C trafficking revealed through the study of a pathogenic mutant

Jennifer A. Dickens[1], Eimear N. Rutherford[1], Susana Abreu[1], Joseph E. Chambers[1], Matthew O. Ellis[1], Annemarie van Schadewijk[2], Pieter S. Hiemstra[2] and Stefan J. Marciniak[1]

[1]Cambridge Institute for Medical Research, Cambridge, UK. [2]Dept of Pulmonology, Leiden University Medical Center, Leiden, The Netherlands.

Corresponding author: Jennifer Dickens (jac72@cam.ac.uk)

Check for updates

Shareable abstract (@ERSpublications)
Studying how SFTPC mutations cause familial pulmonary fibrosis may help us develop new therapies for IPF. By studying a pathogenic mutation, this study reveals new insights into SFTPC handling in alveolar cells and how this is perturbed in disease. https://bit.ly/344VUhm

Cite this article as: Dickens JA, Rutherford EN, Abreu S, *et al*. Novel insights into surfactant protein C trafficking revealed through the study of a pathogenic mutant. *Eur Respir J* 2022; 59: 2100267 [DOI: 10.1183/13993003.00267-2021].

This single-page version can be shared freely online.

Copyright ©The authors 2022.

This version is distributed under the terms of the Creative Commons Attribution Licence 4.0.

This article has supplementary material available from erj.ersjournals.com

This article has an editorial commentary: https://doi.org/10.1183/13993003.02147-2021

Received: 28 Jan 2021
Accepted: 16 May 2021

Abstract

Background Alveolar epithelial cell dysfunction plays an important role in the pathogenesis of idiopathic pulmonary fibrosis (IPF), but remains incompletely understood. Some monogenic forms of pulmonary fibrosis are associated with expression of mutant surfactant protein C (SFTPC). The commonest pathogenic mutant, I73T, mislocalises to the alveolar epithelial cell plasma membrane and displays a toxic gain of function. Because the mechanisms explaining the link between this mutant and IPF are incompletely understood, we sought to interrogate SFTPC trafficking in health and disease to understand the functional significance of SFTPC-I73T relocalisation.

Methods We performed mechanistic analysis of SFTPC trafficking in a cell model that reproduces the *in vivo* phenotype and validated findings in human primary alveolar organoids.

Results We show that wild-type SFTPC takes an unexpected indirect trafficking route *via* the plasma membrane and undergoes the first of multiple cleavage events before reaching the multivesicular body (MVB) for further processing. SFTPC-I73T takes this same route, but its progress is retarded both at the cell surface and due to failure of trafficking into the MVB. Unable to undergo onward trafficking, it is recycled to the plasma membrane as a partially cleaved intermediate.

Conclusion These data show for the first time that all SFTPC transits the cell surface during normal trafficking, and the I73T mutation accumulates at the cell surface through both retarded trafficking and active recycling. This understanding of normal SFTPC trafficking and how the I73T mutant disturbs it provides novel insight into SFTPC biology in health and disease, and in the contribution of the SFTPC mutant to IPF development.
